# The Links between ALS and NF-κB

**DOI:** 10.3390/ijms22083875

**Published:** 2021-04-08

**Authors:** Emma Källstig, Brian D. McCabe, Bernard L. Schneider

**Affiliations:** Brain Mind Institute, Ecole Polytechnique Fédérale de Lausanne (EPFL), Station 19, 1015 Lausanne, Switzerland; emma.kallstig@epfl.ch (E.K.); brian.mccabe@epfl.ch (B.D.M.)

**Keywords:** ALS, neuroinflammation, NF-κB

## Abstract

Amyotrophic lateral sclerosis (ALS) is a neurodegenerative disease wherein motor neuron degeneration leads to muscle weakness, progressive paralysis, and death within 3–5 years of diagnosis. Currently, the cause of ALS is unknown but, as with several neurodegenerative diseases, the potential role of neuroinflammation has become an increasingly popular hypothesis in ALS research. Indeed, upregulation of neuroinflammatory factors have been observed in both ALS patients and animal models. One such factor is the inflammatory inducer NF-κB. Besides its connection to inflammation, NF-κB activity can be linked to several genes associated to familial forms of ALS, and many of the environmental risk factors of the disease stimulate NF-κB activation. Collectively, this has led many to hypothesize that NF-κB proteins may play a role in ALS pathogenesis. In this review, we discuss the genetic and environmental connections between NF-κB and ALS, as well as how this pathway may affect different CNS cell types, and finally how this may lead to motor neuron degeneration.

## 1. Introduction

Amyotrophic lateral sclerosis (ALS), also known as Lou Gehrig’s disease, Charcot disease or sometimes Motor Neuron Disease (MND), is a neurodegenerative disorder described in 1874 by French neurologist Jean-Martin Charcot [1]. It affects the motor system and leads to degeneration of motor neurons in the cerebral motor cortex, in addition to the brainstem and spinal cord [2]. The consequent disruption of communication between the nervous system and skeletal muscles initially results in a characteristic pattern of muscular weakness and spasticity [3]. As the denervation of muscles advances, patients develop paralysis, and a majority will die due to respiratory failure within 3–5 years of diagnosis.

Today, the annual incidence of ALS in a general European population is measured as 2.16 cases per 100,000 people and is projected to increase in the future [4,5]. Approximately 10% of ALS cases are attributed to inherited familial mutations in more than 25 different genes including: Chromosome 9 Open Reading Frame 72 (c9orf72), Superoxide Dismutase 1 (SOD1), TAR DNA-binding protein 43 (TDP-43), Fused in Sarcoma (FUS), Optineurin (OPTN), and TANK-binding kinase 1 (TBK1) [6]. The remaining majority of cases are considered as “sporadic” and their etiology is still poorly defined.

Neuroinflammation has become increasingly recognized as an important factor underlying the cell-specific neurodegeneration observed in many diseases of the central nervous system (CNS), including ALS. Elevated levels of proinflammatory cytokines as well as activation of microglia and astrocytes have been observed in Alzheimer’s disease, Parkinson’s disease and Multiple Sclerosis, to name a few [7,8,9]. As for these diseases, the increasing recognition of the potential role of neuroinflammation in ALS has promoted expanded investigation, and several studies have proven the presence of inflammation in ALS patients, an observation also made early in the disease process in ALS animal models [10,11,12]. Moreover, levels of the inflammatory factors correlate with the severity of the disease [13]. Both astrocytes and microglia are known to be the main sources of the inflammatory mediators that control immune responses in the CNS. Therefore, these cell types have been put under particular scrutiny. In the high-copy SOD1^G93A^ mouse model of ALS, disease symptoms onset at around 3–4 months and progress until death occurs at the age of 5–6 months [14]. In this model, microglial activation has been observed from 80 days of age up to end stage. Astroglial activation has been observed from day 100 with differing results found for the activation level at 120 days [15,16]. In diverse studies of ALS models and patients, astrocytes and microglia have both been shown to produce an increase in activation of the nuclear factor kappa-light-chain enhancer of activated B cells (NF-κB) [17,18]. The NF-κB signaling pathway has an important role in the induction of pro-inflammatory gene expression, this further supporting a role for neuroinflammation. In this review, we will explore the connection between NF-κB and ALS, including within the many different cell types that could contribute to the degeneration of motor neurons.

## 2. NF-κB Signaling

### 2.1. NF-κB Mechanism of Action

**Figure 1:**
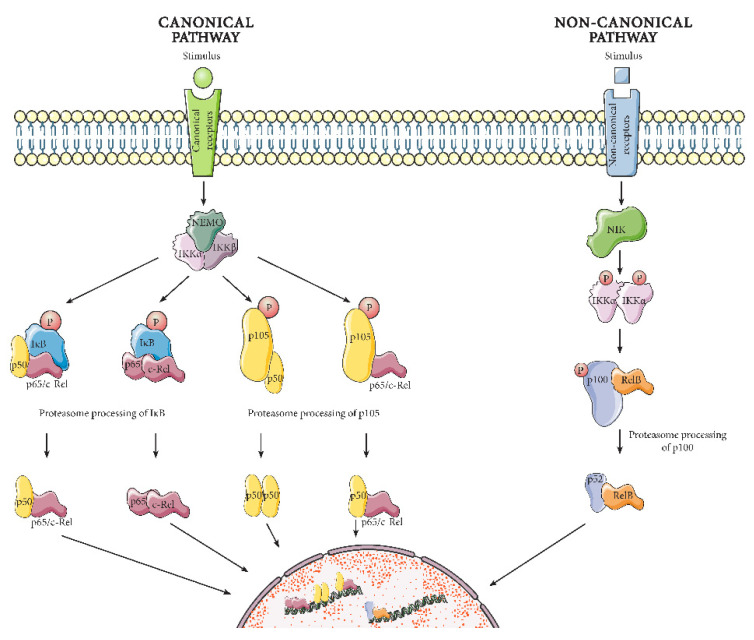
Simplified illustration of the canonical and non-canonical versions of the NF-κB pathway. When a stimulus activates a canonical pathway receptor, the IκB kinase (IKK) complex forms. The IKK complex phosphorylates the inhibitory IκB protein and the p50 precursor protein p105, inducing their degradation and processing, and enabling the translocation of the NF-κB dimers into the nucleus for gene expression regulation. Upon activation of the non-canonical pathway, NF-κB-inducing kinase (NIK) activates the IKKα dimer, leading to phosphorylation and proteasome processing of p100. This results in production of p52, which together with RelB translocates to the nucleus and regulates transcription. The dimers presented in this figure are not the only possible combinations.

NF-κB was discovered by Sen and Baltimore in 1986, as a protein present in activated B lymphocytes that binds to a specific, conserved DNA sequence [19]. Subsequently, NF-κB has been shown to be part of a highly conserved protein family, present in organisms as diverse as *Drosophila melanogaster* [20], mice and humans [21]. NF-κB members act as constituents of related protein complexes [22]. The family has five members: p50, p52, p65/RelA, c-Rel, and RelB, which all share the presence of a Rel homology domain adjacent to a nuclear localization signal. The Rel homology domain enables DNA binding, interactions with IκB inhibitory proteins, and dimerization. The NF-κB pathway must be activated to recruit the NF-κB proteins in functional complexes and induce transcription. As shown in Figure 1, there are two main pathways to NF-κB activation: the canonical and the non-canonical [23]. The canonical pathway responds to several different immune receptors, predominantly resulting in the activation of NF-κB proteins p65/RelA, p50, and c-Rel. The non-canonical pathway, on the other hand, is triggered primarily by a certain subset of receptors from the tumor necrosis factor receptor (TNFR) superfamily, causing induction of p52 and RelB. Below, we will discuss further the differences between the two pathways, as well as their diverse activities. It is however worth noting that the canonical and non-canonical pathways are not mutually exclusive. For example, canonical p65/p50 dimers can be induced by the non-canonical pathway [24]. In addition, the p65/p50 dimer itself can also regulate parts of the non-canonical pathway. The full extent of this interdependency is not yet completely understood.

#### 2.1.1. Canonical Pathway

The canonical pathway (hereafter known as the NF-κB pathway) responds to immediate infectious threats and pro-inflammatory signals [23,25]. In the absence of such activating threats, the NF-κB proteins p65, p50 and c-Rel are located in the cytoplasm. There, they are bound to Inhibitor of κB (IκB) proteins or the IκB-like p50 precursor protein, p105. These inhibitors prevent nuclear translocation of the NF-κB proteins by masking their nucleus localization signal. When an infection or pro-inflammatory stimulus reaches the cell, many different receptors can serve as activators of the pathway including pattern-recognition receptors, TNF receptor superfamily members, T-cell receptors, B-cell receptors in addition to several cytokine receptors [26]. Activation of these receptors initiates a cascade of intracellular proteins that induces the formation of the IκB kinase (IKK) complex. The IKK complex contains a regulatory subunit called NF-κB regulatory modifier (NEMO) that tethers the two other subunits, the kinases IKKα and IKKβ. The IKK complex phosphorylates the inhibitory IκB proteins and p105, which leads to their degradation or processing by the proteasome. With the NF-κB protein dimers now freed from inhibition, they transfer to the nucleus where they activate or repress inflammatory gene transcription.

#### 2.1.2. Non-Canonical Pathway

The non-canonical pathway is regulated via a different protein complex and relies on protein synthesis [27]. Hence, its activation is typically much slower, and plays roles during immune cell differentiation and development [23,25,28]. Much like the canonical pathway, in the absence of activation the non-canonical pathway NF-κB proteins RelB and p52 precursor p100 are bound to each other in the cytoplasm. In this situation, p100 functions similarly to an IκB protein by inhibiting the nuclear translocation of RelB. In the presence of non-canonical receptor stimulation (through certain TNF receptor superfamily members such as CD40, lymphotoxin β receptor, B-cell activating factor receptor, etc.), the NF-κB-inducing kinase (NIK) activates downstream IKKα dimers, which in turn phosphorylate p100 and mark it for proteasome processing. Processed p100, also known as p52, no longer inhibits the nuclear localization of RelB. The RelB/p52 heterodimer translocates to the nucleus where it regulates gene expression.

### 2.2. Functions of NF-κB

The NF-κB pathway is firmly established for its critical role in the immune system. Upon their initial discovery, NF-κB proteins were viewed as inducers of B cell development and activation but have since then also been shown to be immediate activators of the innate immune system at the sites of an infection or wound [29]. In the case of an extensive inflammation, the NF-κB proteins will also contribute to the activation of the adaptive immune system to keep the infection at bay. NF-kB proteins also have other peripheral functions that could have consequences for ALS disease progression. For example, the NF-kB pathway has been found to have important roles in muscle tissue, where overactivation results in muscle atrophy [30]. At the neuromuscular junction (NMJ), the NF-kB pathway also contributes to acetylcholine receptor clustering [31]. Whether activation of NF-kB in muscles could be involved in ALS progression has yet to be determined. However, several observations suggest that peripheral immune cells could play important roles in neuromuscular diseases. For example, peripheral macrophages surrounding motor neuron axons are activated in both ALS mouse models and ALS patients [32]. In the SOD1^G93A^ mouse model, replacing peripheral macrophages with macrophages that have been genetically modified to reduce reactive oxygen species leads to decreased activation of both the peripheral macrophages and of CNS microglia when carried out at disease onset. The effects of the immune response in these peripheral cells upon neuromuscular disease progression continues to be explored [33].

In addition to these essential roles in other tissues, the NF-κB pathway is also directly involved in CNS function. Several NF-κB transcription factors are expressed in neurons, including the p50-p65 heterodimer and the p50-p50 homodimer. These NF-κB proteins can mediate transcriptional response to synaptic activity and play key roles in learning and memory [34]. Furthermore, NF-κB proteins have been found not only in the neuronal soma, but also in neuronal processes and synapses, where the p50-p65 heterodimer has been singularly observed. In p65-deficient mice, NF-κB dimers were no longer found in synapses whereas other NF-κB proteins were still present in the cell bodies, implying a particular role for the p65-p50 dimers in synapses. In a similar way to activation in the periphery, in the CNS the NF-κB pathway can be triggered by cytokines, interleukins, viral infections, and oxidative stress [35]. However, these inducers may not induce the same activity in the CNS as in the periphery. For example, the cytokine tumor necrosis factor α (TNFα) is well known to be involved in the tissue injury response in peripheral tissues. However, in the CNS, in the absence of inflammation, it can also mediate neural plasticity in the hippocampus [36]. Similarly, the free radical nitric oxide regulates synaptic efficacy in the CNS, while in the peripheral immune system it has a role in cell death [37]. Given the many roles of the NF-κB pathway in both the nervous system and the immune system, disentangling how these many functions could impact the progression of neurodegenerative diseases including ALS remains a serious challenge.

## 3. ALS Genetics

**Figure 2:**
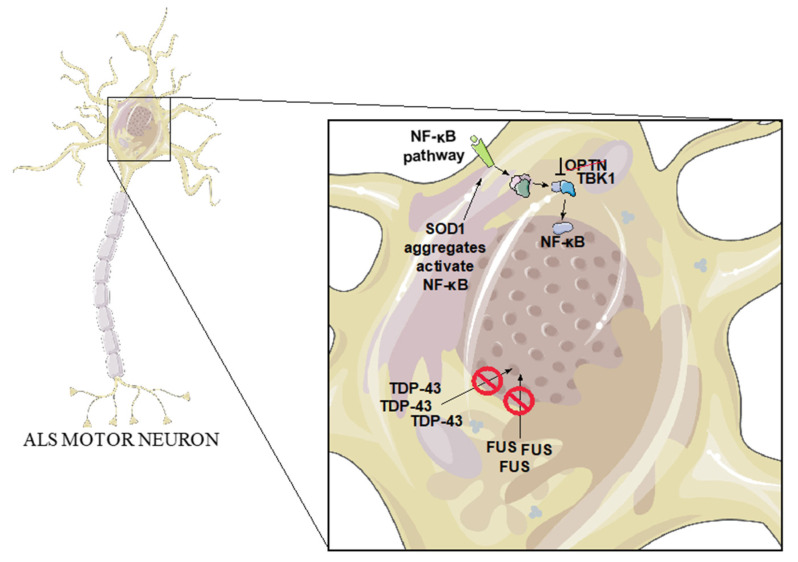
Schematic representation of a motor neuron showing how major genetic risk factors linked to amyotrophic lateral sclerosis (ALS) may affect the NF-κB pathway. The NF-κB pathway can be activated by either SOD1 protein aggregation or other stimuli. OPTN has limited ability to inhibit the NF-κB pathway when carrying loss-of-function ALS mutations. Mutated TBK1 is unable to bind to OPTN but may still be regulating the NF-κB pathway. Both TDP-43 and FUS, which usually bind to NF-κB in the nucleus, accumulate in the cytoplasm and may fail to maintain their nuclear activity in conditions leading to ALS.

In order to explore possible interaction of the NF-κB pathway in ALS, one first approach is to identify associations with genetic forms of the disease. The 10% of ALS cases that are of familial genetic origin can provide insight into the molecular mechanisms that may also be at play in the remaining 90% of sporadic cases [38]. Below, we will discuss a selection of some of the over 25 genes which have a genetic link to ALS, focusing on those with a potential connection to neuroinflammation and NF-κB activity (Figure 2). In addition to this selected subset, it should be noted that several other familial ALS genes have a connection to NF-κB and inflammation, such as Ubiquilin-2 [39] and Sequestosome 1 [40].

### 3.1. C9orf72

Expansion of a GGGGCC hexanucleotide repeat in the first intron of the c9orf72 gene is the most common genetic defect associated with familial ALS (fALS), causing 39% of fALS cases and 7% of sporadic ALS (sALS) cases with European ancestry [41]. In non-diseased individuals, the first intron of the c9orf72 gene contains ≤ 11 hexanucleotide repeats but, in the case of ALS patients, these repeats are expanded, ranging from hundreds to thousands of repeats [42,43]. It has been suggested that these repeats could cause disease via bidirectional transcription into RNAs which form unusual secondary structures [44]. Another potential pathogenic effect is repeat-associated non-ATG (RAN) translation into dipeptide repeats which accumulate in diseased cells [45]. An additional and perhaps not mutually exclusive mechanism that could contribute to disease is that the presence of the hexanucleotide repeats in the c9orf72 intron leads to reduced expression of the c9orf72 protein itself. To investigate the possibility that a loss-of-function of c9orf72 contributes to disease, several *c9orf72* null mutant mouse strains have been developed. These mutants show mild or no motor phenotypes, but instead have increased immune response activation [46,47]. For example, in a study by O’Rourke et al., c9orf72^−/−^ mice showed progressive accumulation of macrophage-like cells [48]. Moreover, the expression of c9orf72 was found to be highest in myeloid cells, and its removal leads to altered immune responses in macrophages and microglia. Additionally, the animals show age-related neuroinflammation. These studies suggest that c9orf72 plays an important role in normal myeloid cell function. Consistently, it was observed that knocking out c9orf72 solely in myeloid cells could mimic the phenotype seen in mice with complete c9orf72 knockout [49]. Myeloid cells depleted of c9orf72 are also hyperresponsive to activators of the innate immune response regulator stimulator of interferon genes (STING) and display reduced degradation of STING through the autolysosomal pathway. Furthermore, the systemic inflammation and autoimmunity of *c9orf72* mutant mice can be reduced when housed in an environment with less immune-stimulating bacteria, further supporting the importance of c9orf72 function in regulating the immune response [50]. Knock-down of c9orf72 activates the NF-κB pathway in particular in a U87 glioblastoma cell model [51], revealing the possibility that NF-κB takes part in c9orf72-dependent immune responses. Altogether, these data support an important role for c9orf72 in innate immune regulation which potentially could contribute to ALS progression.

### 3.2. SOD1

The first gene to be discovered as an ALS-associated gene encodes Superoxide Dismutase 1 (SOD1). Over 170 ALS-causing mutations have been found in *SOD1* [52], and SOD1 aggregation has been observed in both fALS and sALS cases. The normal function of the SOD1 dimeric enzyme is to catalyze the breakdown of superoxide into oxygen and hydrogen peroxide, in order to protect the cell from the toxicity of these reactive oxygen species [53]. Although this process might be disrupted in some of the mutant versions of the gene [52], most experimental evidence supports a gain of toxic activity in SOD1-related ALS pathology. Mutant SOD1 aggregates observed in fALS are, for example, believed to cause neurotoxicity [53]. This is bolstered by the fact that mouse models of ALS based on high-copy overexpression of mutant SOD1 develop a disease very similar to human ALS [14]. As with most neurodegenerative diseases, presence of aggregated proteins has been hypothesized to induce neuroinflammation. Indeed, both microglial and astroglial activation have been observed in SOD1^G93A^ mice [15]. Moreover, Adeno-associated virus (AAV)-mediated silencing of astrocytic SOD1^G93A^ in the SOD1^G93A^ mouse model leads to late-stage rescue of neuromuscular junction (NMJ) occupancy and improved motor behavior [54]. Likewise, diminishing the expression of mutant SOD1 solely in microglia using Cre-dependent transgene inactivation in floxed SOD1^G37R^ mice slows down late disease progression [55]. In addition, SOD1^G93A^ mice lacking myeloid and lymphoid cells have a similar disease progression as SOD1^G93A^ mice but, when given a wild-type bone marrow transplant, disease progression significantly slows down [56]. In vitro, it has also been observed that SOD1^G93A^ microglia release more neurotoxic factors compared to wild-type microglia [56]. When co-cultured with SOD1^G93A^ motor neurons, SOD1^G93A^ microglia induce significantly higher motor neuron death than wild-type microglia. Pro-inflammatory factors such as IL-1α, TNFα and C1qa can be released by neuroinflammatory activated microglia and induce neurotoxicity via changes in astrocyte activity. Indeed, SOD1^G93A^ mice with a triple knockout of IL-1α, TNFα and C1qa show over 50% prolongation of survival and improved motor abilities [57]. These effects are likely to be related to changes in neuroinflammation and thereby could be due to, or lead to, changes in NF-κB activity. Along these lines, NF-κB is activated mainly in microglia in SOD1^G93A^ mice, and deletion of NF-κB signaling in microglia prolongs mouse survival [17]. Similarly, expression of SOD1^G93A^ and SOD1^G85R^ in cell lines is sufficient to trigger activation of NF-κB [58], further suggesting that aggregated SOD1 may lead to immune response activation. Mutated SOD1 also leads to ER stress and activation of the unfolded protein response (UPR), which in turn can activate NF-κB [59], suggesting a possible mechanism for NF-κB activation in ALS.

### 3.3. TDP-43

Approximately 5% of fALS patients have mutations in the *TDP-43* gene, mostly inherited in an autosomal dominant manner [41]. However, up to 97% of ALS patients present with TDP-43 protein aggregation independently of TDP-43 mutations [38], showing the importance of this protein in ALS. Normally, TDP-43 functions as an RNA- and DNA-binding protein regulating transcription, mRNA splicing, transport, and stability. It is predominantly a nuclear protein but in case of ALS TDP-43 is often found to re-localize to the cytoplasm where the protein is found in dynamic stress granules as well as in protein inclusions [60,61]. It is likely that these inclusions can lead to a toxic gain-of-function as they interact with cellular components, but the loss of TDP-43′s nuclear functions may also be a source of neuronal toxicity. A study by Swarup et al. has further illuminated the pathological role of the protein in human ALS spinal cords. TDP-43 and p65 were observed to co-localize and interact in glial cells and neurons, indicating potential interactions of TDP-43 with the NF-κB pathway [62]. In addition, electrophoretic mobility shift assay (EMSA) revealed that interaction between p65 and DNA is significantly increased when co-transfected with TDP-43^WT^ or TDP-43^G348C^, suggesting that TDP-43 could act as a co-activator of p65. Overexpression of TDP-43 in neuronal cell lines exposed to glutamate toxicity also leads to cell death that can be reduced by inhibition of NF-κB activity [62]. It has also been suggested that mutated TDP-43 enters the mitochondria, triggering release of mitochondrial DNA, and thereby causes activation of the NF-κB-activating cGAS/STING pathway, resulting in inflammation [63]. Crossing a mouse model overexpressing human TDP-43^A315T^ to mice deficient in innate immune response regulator STING results in a 40% lifespan prolongation and improves motor abilities, despite not leading to a decrease in TDP-43 expression. Expression of a STING inhibitor in the TDP-43^A315T^ model also improved motor behavior, reduced neuronal death, and decreased expression of NF-κB-induced genes in cortex and spinal cord. In addition, activated microglia and astrocytes have been observed in the motor cortex of both ALS patient samples with TDP-43 pathology and a TDP-43 mouse model, suggesting an inflammatory response in this area [64]. Primary mouse astrocytes transfected with TDP-43 also show increased inflammatory response [65]. In mice with AAV9-mediated neuronal TDP-43 expression, low-dose lipopolysaccharides (LPS) were continually introduced to induce a chronic inflammatory response. These studies show increased permeability of the blood–brain barrier (BBB) in TDP-43-expressing mice, leading to an LPS-induced inflammatory response in the CNS. TDP-43 mice without LPS treatment also displayed infiltration of CD3+, CD4+ T cells, and immunoglobulin G (IgG) through the BBB pointing to a regulatory role of TDP-43 in the immune system [66]. Moreover, cultured microglia and astrocytes expressing either TDP-43 WT or TDP-43^A315T^ and exposed to LPS display increased TDP-43 expression in both genotypes. In addition, both TDP-43^WT^ and TDP-43^A315T^ are mislocalized to the cytoplasm after LPS treatment, mimicking the cytoplasmic presence of TDP-43 in ALS. TDP-43-containing puncta were observed in the cytoplasm, at a higher level in TDP-43^A315T^ than in TDP-43^WT^ cells [67]. In summary, these results suggest a role of TDP-43 in the immune system potentially involving changes in NF-κB activity and suggest the resulting inflammatory response could induce TDP-43 mislocalization.

### 3.4. FUS

FUS is an RNA and DNA binding nuclear protein that can regulate mRNA splicing and gene expression. In around 5% of the fALS cases, mutations are observed in the *FUS* gene [41], many of them disrupting the nuclear localization signal of the protein and causing accumulation of FUS in the cytoplasm [68]. Again, it is unclear whether a gain- or loss-of-function of FUS causes the pathology. FUS^WT^ overexpression in *Drosophila*, mice and rats all led to neurotoxicity [69]. Moreover, mice expressing near endogenous levels of mutant FUS also display ALS-like pathology [70]. However, in *Drosophila melanogaster*, it has also been seen that deletion of the FUS ortholog *Caz* leads to a severe ALS-like phenotype, suggesting that the cause of the pathology may involve loss-of-function [71]. Similar to TDP-43, the function of FUS has been further explored in the context of NF-κB signaling as the FUS protein also interacts with p65. FUS may act as a co-activator of p65 as it is able to enhance the NF-κB response to stimuli such as TNFα, interleukin-1β (IL1β), and overexpression of NF-κB-inducing kinase [72]. In cell lines, it has also been discovered that treatment with immune response protein type I interferon (IFN) promotes FUS mRNA stability leading to FUS accumulation [73]. When overexpressing FUS in vitro in mouse and human astrocytes derived from neural progenitors, the FUS-expressing astrocytes were found to have a higher pro-inflammatory response than controls when stimulated with IL1β [74]. Medium from these astrocytes also promoted microglial activation and neuronal cell death in vitro. Therefore, FUS may regulate the immune response and, when mutated in ALS, may also promote neuroinflammation via NF-κB activity.

### 3.5. OPTN

ALS-causing *OPTN* mutations are most frequent in Japanese and Chinese patients where they are responsible for 3% of the fALS cases, compared to 1% of Caucasian fALS cases [75]. The OPTN protein has many functions: induction of autophagy, structural maintenance of the Golgi complex, regulation of post-Golgi protein trafficking, as well as inhibition of the NF-κB pathway [76]. To inhibit NF-κB, OPTN competes with NEMO for binding to the ubiquitinated receptor-interacting protein kinase 1 (RIPK1). Without binding NEMO, RIPK1 is unable to activate the IKK complex, which prevents NF-κB activation [77]. In ALS patients, OPTN has been observed in protein inclusions, co-localizing with SOD1, TDP-43, or FUS [78]. Interestingly, the *OPTN* mutations associated with ALS lead to autosomal recessive inheritance of the disease, which supports loss-of-function as the most likely pathogenic mechanism [79]. The expression pattern of p65 has been analyzed in patients with sALS or fALS with *OPTN* mutations [80]. p65 was found to be increased in microglia and absent from neuronal nuclei suggesting that neuroinflammation is present and that microglia are activated. Following knock down of OPTN in a neuronal cell line, NF-κB expression and neuronal cell death are increased [78]. The neuronal cell death can be hindered by the NF-κB inhibitor withaferin A. While *Optn* deficient mice develop and breed normally [75], they develop motor axon degeneration and progressive demyelination through CNS cell necroptosis and neuroinflammation [81]. This phenotype can be rescued by blocking cell death via inhibition of the protein RIPK1′s regulation of inflammation. In ALS, mutated OPTN seems to have neurodegenerative effects via loss-of-function possibly leading to dysregulated NF-κB activity, although it is still unclear whether this happens directly in neurons or through non-cell autonomous mechanisms.

### 3.6. TBK1

TBK1 is a tightly regulated kinase, which is involved in many different pathways such as autophagy, cell proliferation, and insulin signaling [82]. It also plays an important role in immunity as a response to viral infection, phosphorylating Interferon regulatory factor 3 (IRF3), which can induce an antiviral gene expression response [83]. Additionally, as its name suggests (TANK-binding kinase 1), it binds and activates the NF-κB regulator TANK (TRAF family member-associated NF-kappa-B activator), thereby regulating the non-canonical NF-κB response [84]. Furthermore, TBK1 binds to and interacts with the previously mentioned NF-κB-inhibitor OPTN [85]. Numerous fALS-associated nonsense mutations have been discovered in TBK1, suggesting haploinsufficiency as the most likely disease mechanism [86]. In addition, several missense mutations have been found, which appear to eliminate TBK1 activity by preventing its homodimerization and abolish its ability to interact with OPTN and Interferon regulatory factor 3 (IRF3). These results suggest a loss-of-function mechanism for TBK1 in ALS pathology, although not all mutations have been characterized yet. Moreover, whereas *Tbk1* knockout selectively in motor neurons does not cause any neurodegenerative phenotype per se, it was found to worsen motor behavior, accelerate denervation of neuromuscular junctions and disease onset when these mice were crossed with SOD1^G93A^-expressing mice [87]. Mice with knock-in of ALS-related TBK1 loss-of-function mutations, in combination with SOD1^G93A^ expression, display decreased levels of interferon-inducible genes in glia, accelerated disease onset, but extended lifespan [87]. Similar results have been seen when introducing a heterozygous *Tbk1* deletion in SOD1^G93A^ mice, which leads to accelerated onset of disease but decreases microglial neuroinflammation and prolongs survival at later disease stages [88]. These results highlight the importance of cell type specificity and timing in future treatments as responses may differ as a function of disease stages and targeted cell types.

## 4. ALS Environmental Factors

While fALS cases can give important clues about ALS molecular mechanisms, it is also important to study environmental factors that could contribute to ALS. Tobacco smoking, for example, has been shown to increase the risk of ALS in several studies, in particular for women [89,90]. Physical head trauma is another ALS risk factor [91]. However, head trauma can also lead to neurodegeneration in the absence of ALS, such as in the case of chronic traumatic encephalopathy (CTE) [92]. It is also worth noting that it is not certain if the physical trauma itself is a cause of the disease in the case of ALS, or if head trauma could be an early symptom of muscle weakness in as yet undiagnosed ALS patients [93]. Physical exercise may also increase the risk of ALS, but not in a dose-dependent manner [94]. Therefore, it has been suggested that exercise itself may not cause disease, but that perhaps a genetic predisposition to exercise is somehow linked to a genetic predisposition to ALS. Furthermore, exposure to metals such as lead have been suggested to be a risk factor. In a study by Fang et al., an association was found between lead levels in the blood and ALS [95]. While none of the above-mentioned risk factors are linked directly to an increasing CNS NF-κB response, they all may expose the body to some type of stress. The consequences of this are not yet completely understood, but it is known that different types of stress can cause inflammation [96].

However, other environmental ALS risk factors have more direct links to neuroinflammation and NF-κB signaling. Several pesticides, for example, have shown an association with ALS. One of these, organophosphates, can cause neurological damage, oxidative stress, and mitochondrial dysfunction [97]. Acute organophosphate intoxication leads to an increase in microglial and astroglial activation, causing a release of pro-inflammatory cytokines [98].

In addition, dietary choices can have an influence on the risk of ALS. Increased intake of antioxidants has been associated with decreased risk of disease, suggesting that oxidative stress may play an important role in disease development [99]. NF-κB signaling has indeed been demonstrated to be activated by oxidative stress, and antioxidant treatment can block this activation [100]. Indeed, inflammatory stimulation induces NF-κB activity in primary culture cortical astrocytes, a change that can be prevented by antioxidant treatment [101]. In primary cortical neurons exposed to the pro-inflammatory astrocyte medium, NF-κB binding to DNA decreased. This, too, could be averted through exposure to antioxidants. Similar results have been observed in primary cultures of rat neuronal cultures [102]. The neurons were exposed to injury via chemical ischemia, resulting in an increased NF-κB activation that could be prevented by administration of antioxidants or an NF-κB inhibitor. These results point to a connection between oxidative stress and NF-κB that could affect inflammation.

Another risk factor of ALS is the neurotoxin β-N-methylamino-L-alanine (BMAA) [103]. BMAA was first linked to ALS when it was discovered in the diet of patients in the Pacific island of Guam, which had a significantly higher incidence of ALS than the rest of the world in the 1950s. BMAA has since been proven to activate the glutamate receptors NMDA and AMPA. This in turn disrupts mitochondrial function, increases reactive oxygen species (ROS) production, and causes apoptosis. A study by Silva et al. proposes a novel mechanism for BMAA in Alzheimer’s disease where the mitochondrial toxicity of BMAA activates Toll-like receptors (TLRs) causing the activation of p65 NF-κB [104]. Indeed, in primary cortical neurons subjected to BMAA, an increase in cell surface TLR4 is observed. A decrease in IκBα causing a translocation of p65 NF-κB to the nucleus is also seen. This causes an increase in the transcription of pro-inflammatory Nod-like receptor 3 (NLRP3) as well as Interleukin 1 beta (IL-1β) precursor pro-IL-1β in the neurons. A similar mechanism could be plausible for BMAA-induced ALS cases. Next, we delve further into another environmental factor that is present in numerous ALS cases and that is known to activate both NF-κB and inflammation—bacterial infection.

### Bacterial Infections in ALS

Bacterial DNA and prokaryotic cells have been observed in CNS tissue of ALS patients [105], and in several cases of ALS, patients have been diagnosed with a bacterial infection. All but one of the Gulf War veterans that were diagnosed with ALS, for example, had systemic *Mycoplasma fermentas* infections [106]. In a cohort of non-military American, Canadian, and British ALS patients, 80% had infections with *M. penetrans, M. fermentans* (59%), *M. hominis*, or *M. pneumoniae*. In addition, studies have shown that the spirochete bacteria, *Borrelia burgdorferi*, is present in a significant proportion of ALS patients [107,108]. Moreover, spirochete bacteria in general have been observed in cases of both ALS and Alzheimer’s disease [109,110]. Mycoplasmas identify neither as Gram-negative nor as Gram-positive, due to their lack of cell wall structure, preventing a Gram’s stain [111]. However, the aforementioned Mycoplasma strains all activate the NF-κB pathway, as do spirochete bacteria [112].

Some researchers explain this connection between bacterial infection and ALS by the so-called endotoxin theory, suggesting that Gram-negative bacteria, in particular, are contributors to neurodegeneration via exposure to the lipopolysaccharide (LPS) endotoxin, the main component of the Gram-negative bacterial outer membrane [113]. Normally, the endotoxin levels in blood plasma are low, but they could be increased during the course of neurodegenerative diseases [114]. When wild-type rodents are injected with high levels of endotoxins, this induces microglial activation, memory deficits and neuronal loss [115,116]. One puzzle is how endotoxin can influence the brain. It has been suggested that LPS may activate cells at the level of the blood–brain barrier, resulting in a release of pro-inflammatory cytokines and recruiting immune cells in the brain [113]. Another possibility is that LPS activates the brain’s immune response through the circumventricular organs. When the CNS is reached, endotoxin causes an inflammatory response by activating Toll-like receptor 4 (TLR4), leading to activation of the NF-κB pathway. The NF-κB pathway in turn induces expression of many pro-inflammatory genes, activating the immune system to clear the bacterial infection and the LPS from the body. However, if LPS is not properly cleared from the blood, this leads to a state of chronic inflammation [117]. When the immune system of the brain is activated, there are several mechanisms that could lead to neurodegeneration, for example via activation of microglial cells which can result in phagocytosis of neurons, including healthy neurons [113].

## 5. The Effect of NF-κB Activation in Different CNS Cell Types during ALS

**Figure 3:**
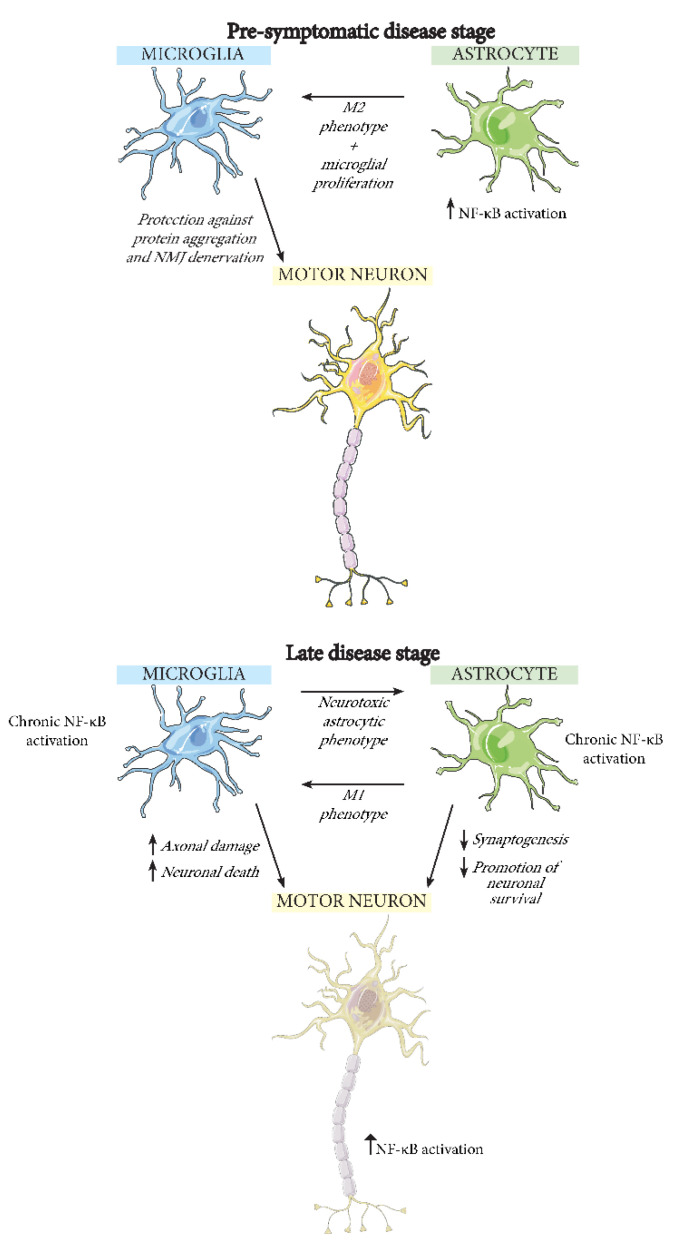
Illustration of the possible interactions between microglia, astrocytes, and neurons, during early and late stage ALS. The NF-κB pathway is activated in both astrocytes and microglia in ALS, but its effects differ between the stages of the disease. Pre-symptomatically, astrocytes with activated NF-κB promote neuroprotective M2-type microglial proliferation, while at a later stage a neurotoxic M1-type microglial phenotype is induced. Subsequently, M1 microglia cause axonal damage and promote motor neuron death. In addition, M1 microglia induce a neurotoxic astrocytic phenotype, leading astrocytes to decrease their synaptogenesis and promotion of neuronal survival.

As has been highlighted in this review, there are several connections between neuroinflammation, in particular involving NF-κB, and ALS. However, there is still the question of how NF-κB activation and inflammation could lead to motor neuron death. Many studies have suggested that motor neuron death is not necessarily cell autonomous and may have involvement from numerous cell types in the CNS. Knock-down of CD8+ T cells in SOD1^G93A^ mice, for example, significantly decreases the spinal motor neuron loss [118]. Macrophages, B cells and natural killer cells are all also present in the CNS [119] and may all affect NF-κB-driven neuroinflammation in ALS. In this review, however, we will focus on exploring the role of NF-κB in neurons, astrocytes, and microglia during ALS (Figure 3).

### 5.1. Neurons

In addition to its function in inflammation, the NF-κB signaling pathway has many other important roles including in neurons. Several studies have made a link between NF-κB and regulation of cognitive behaviors such as learning and memory [120,121,122]. When activated in mouse excitatory glutamatergic neurons, NF-κB has also been observed to stimulate excitatory synapse formation [123]. This effect was seen to be reversed when NF-κB signaling was decreased, resulting in reduced dendritic spine size and density, both during development as well as in mature neurons in conditions where there is increased demand for new synapses. NF-κB has also been observed to be activated by excitatory neurotransmitters in mouse cerebellar development [124]. Given these important functions of transient NF-κB activation in synaptic plasticity, development, and neuronal activity [125], it is not implausible to suggest that conditions producing chronic NF-κB activation could instead lead to neurodegeneration. As NF-κB has been shown to be upregulated in the spinal cord of ALS patients [62], it is possible that the activity of this pathway in neurons could contribute to the disease in different ways. For example, a study by Dutta et al. demonstrated that inhibition of the NF-κB pathway through neuron-specific expression of a super-repressor form of IκBα in both TDP-43^A315T^ and TDP-43^G348C^ transgenic mice showed improvements of motor and cognitive functions and reduction in motor neuron loss [126]. In SOD1^G93A^ mice, the same NF-κB inhibitory approach showed increased lifespan and a decrease in misfolded SOD1 accumulation. Collectively, these studies point to an important role of NF-κB in neuronal development and function and highlight the possibility that chronic NF-κB activation contributes to neuronal degeneration in ALS.

### 5.2. Microglia

Microglia normally exist in the brain in a resting state, but they become activated when subjected to an immune challenge or injury [125]. Studies by Kyrargyri et al. looked into the function of NF-κB in microglia when they are not subjected to an immune stimulus [127]. They used mice with partial microglial depletion of IKKβ, and thereby decreased NF-κB activity, and showed that the mice have problems with hippocampus-dependent learning. This suggests microglial NF-κB is not only important in inflammation. Still, NF-κB activity in microglia is most commonly studied in response to an immune challenge, where their activation induces expression of pro-inflammatory mediators [125]. Such microglial activation has, as previously mentioned, been observed in SOD1^G93A^ mice after day 80. In addition, it has been observed in the brains of sporadic ALS patients through positron emission tomography (PET), between 10 and 44 months after first disease symptoms [128]. There exist two types of activated microglia: M1 and M2 [129]. When M1 microglia are stimulated by pathogenic or pro-inflammatory molecules, this induces release of several pro-inflammatory mediators. M2 microglia, on the other hand, are induced by anti-inflammatory cytokines and typically suppress inflammation and promote neuronal survival. In older mice, CNS microglia have more prominent M1 microglia while younger mice have more M2 microglia [130]. In SOD1^G93A^ mice, several of the toxic M1 microglial factor levels are increased [131], and it is noteworthy that M1 microglial activation can be induced by exposure to LPS or pro-inflammatory cytokines [132]. Chronic activation of NF-κB has also been shown to promote the M1 microglial activation and can lead to motor neuron death [17]. In vitro, SOD1^G93A^ microglia have been demonstrated to induce axonal damage and motor neuron death, as well as performing phagocytosis of leftover neuronal debris. This neuronal death can be rescued by depletion of microglial NF-κB signaling and the survival of ALS model mice can be prolonged due to the decrease in inflammatory microglial activation [17].

### 5.3. Astrocytes

The normal role of NF-κB signaling in healthy astrocytes remains understudied. However, a study by Zhang et al. has demonstrated that astrocytic NF-κB upregulation in the hypothalamus leads to glucose intolerance, blood pressure rise, and body weight increase, suggesting a role of NF-κB in the astrocytic control of these factors [133]. With respect to ALS, it is interesting to note that astrocyte-mediated clearing of glutamate from the synaptic cleft is dependent on astrocytic NF-κB activation, a process controlled by neurons [134].

However, the most explored function of NF-κB in astrocytes is related to disease conditions. In particular, exposure of these cells to pathogens leads to increased expression of pro-inflammatory genes to combat infections [135]. This pro-inflammatory astrocyte phenotype is suggested to be induced by NF-κB [136] and leads to failure of many astrocytic neuroprotective qualities, such as promotion of neuronal survival and synaptogenesis [137]. Co-culturing of pro-inflammatory astrocytes with spinal motor neurons produces greatly reduced neuronal viability, an effect that has been attributed to the possible release of a neurotoxin that remains to be characterized. This pro-inflammatory astrocytic phenotype can be activated by microglia, but astrocytic NF-κB activation can also itself in turn be an activator of microglia [138].

In ALS, the interplay between astrocytes and microglia could affect disease progression. For example, diminishing the expression of mutant SOD1^G37R^ selectively in astrocytes delays microglial activation and slows late disease progression in a mouse model of ALS, suggesting that astrocytes take part in regulation of microglial activation [139]. In SOD1^G93A^ mice, astrocytes with activated NF-κB pathway are able to induce neuroprotective microglial proliferation at pre-symptomatic stages, leading to decreased neuronal SOD1 aggregation, retarded NMJ denervation, and thereby deceleration of early disease progression [138]. However, in later stages of the disease, the astrocytic NF-κB pathway activates M1-like microglia, which accelerates the disease progression. These results point to the complex roles of glial cells with differing effects depending upon disease stage. Disappointingly however, in two separate studies, inhibition of the NF-κB pathway selectively in astrocytes has not led to any improvement in the pathology of SOD1^G93A^ mice [17,18]. This may suggest that any possible astrocytic NF-κB-regulating therapeutic for ALS may not only require precise timing of its activity but perhaps also require co-manipulation of microglia.

## 6. Conclusions

The association of neuroinflammation with ALS is established and the role of the NF-κB immune pathway in disease pathophysiology is receiving increasing focus. Direct connections with NF-κB signaling, be it activation, inhibition or binding, have been observed with several genetic and environmental risk factors for ALS. Furthermore, NF-κB signaling is activated in several cell types in ALS models. Inhibition of NF-κB in microglia in particular leads to increased motor neuron survival and slower disease progression. Beneficial effects have also been shown for neuron-specific NF-κB inhibition. However, downregulation of NF-κB signaling in astrocytes has so far shown limited effects.

In light of these studies, when considering the mechanisms of NF-κB signaling in ALS pathophysiology or future potential therapeutics, it is important to contemplate cell type context, the complicated network of aberrant molecular communication between cells that could contribute to motor neuron degeneration, and the temporal state of disease progression. Astrocytic NF-κB activation early in the course of disease, for example, can be protective. Later in the disease progression, however, it can accelerate disease via activation of microglia. Nonetheless, the deep web of links we have reviewed here between NF-κB signaling with both genetic and environmental risks for ALS, should support the continued investigation of manipulating this pathway as a potential intervention to treat this devastating disease.
